# Evaluation of Correlation between the In Vitro Susceptibility of Field Isolates of *Leishmania major* and Clinical Outcomes of Meglumine Antimoniate Therapy in Fars Province, Iran

**Published:** 2017-03-14

**Authors:** Nafiseh Ghobakhloo, Mohammad Hossein Motazedian, Behrad Pourmohammadi, Zahra Yousefi

**Affiliations:** 1 Department of Parasitology and Mycology, School of Medicine, Shiraz University of Medical Sciences, Shiraz, Iran; 2 Student Research Committee, Shiraz University of Medical Sciences, Shiraz, Iran; 3 Basic Sciences in Infectious Diseases, Research Center, Shiraz University of Medical Sciences, Shiraz, Iran; 4 Department of Public Health, School of Health, Semnan University of Medical Sciences, Semnan, Iran

**Keywords:** In vitro susceptibility, Antimonial resistance, *Leishmania major*, Iran

## Abstract

**Background:**

This study was designed to detect whether there is a correlation between in vitro susceptibility of field isolates of *Leishmania major* and the clinical outcomes of meglumine antimoniate (Glucantime®) therapy, the mainstay of cutaneous leishmaniasis treatment in Iran.

**Methods:**

Forty-three patients infected with *L. major* were enrolled in this study from October 2009 to March 2010 and categorized as responsive or unresponsive to Glucantime® treatment after receiving the appropriate therapy. Then, intracellular amastigote approach was conducted on these field strains to investigate in vitro drug susceptibility as well.

**Results:**

At clinical level, out of 43 patients, 15 were clinically non-responsive and 28 were responsive to antimony therapy. All those 28 clinically sensitive strains were susceptible to antimony in the in vitro assay, whereas merely 11 isolates from 15 non-healing isolates were resistant in vitro. Finally, a good correlation (78.9%) with high sensitivity, specificity (100/73) between clinical outcomes and the in vitro susceptibility test was achieved.

**Conclusion:**

The intracellular amastigote model could be an appropriate assay for evaluation of the in vivo drug sensitivity of field isolates. However, more comprehensive studies with larger sets of isolates are needed to confirm these preliminary data.

## Introduction

The most common form of leishmaniasis, Zoonotic Cutaneous leishmaniasis (ZCL), caused by *L. major*, is found in many regions of the middle east including Iran (Razmjou et al. 2009, Jacobson 2011, Alvar et al. 2012, Akhoundi et al. 2013). Since their discovery over 6 decades ago, the antimonial compounds have played vital roles in treatment of all forms of leishmaniasis (Croft et al. 2006). The emergence of antimony resistance as the mainstay of treatment, however, has represented critical health problems in most endemic areas including Iran (Hadighi et al. 2006, Sundar and Goyal 2007, Pourmohammadi et al. 2011, Mohammadzadeh et al. 2013). Since there is no effective vaccine for prevention, disease control is essentially based on chemotherapy.

As resistance phenomenon can cause serious effects on disease treatment and control, this issue is an urgent concern (Sundar et al. 2014). Hence, estimating the efficacy of standard drugs and determining the prevalence of resistance in endemic regions seem to be extremely essential (Croft 2001). Unfortunately, there is no applicable in vitro test for evaluating the clinical resistance. In recent years, some promastigote and intracellular amastigote assays have been established to investigate the effectiveness of anti-*Leishmania* drugs. Fumarola et al. (2004) for screening new compounds against *L. infantum* investigated intracellular amastigotes than promastigotes. Sereno et al. (2007) used green fluorescent protein or luciferase for screening the drug resistance. An in vitro intracellular model for screening the *Leishmania* field isolates were applied by da Luz et al. (2009) and they mentioned that this model needed to be evaluated and standardized for the field isolates. An intracellular model for drug screening also was recommended by Vermeersch et al. (2009) using reference strain of *L. donovani* in different in vitro models. Regardless of the advantages and disadvantages mentioned for these in vitro methods, the intracellular amastigote model is a more reliable in vitro assay for measuring treatment failure in the field isolates (Maia et al. 2013).

In this context, there are some previous reports on clinical evaluation of drug sensitivity, but these studies did not use the standard drug dosage as recommended by WHO and a few studies have been conducted to evaluate the in vitro susceptibility of *Leishmania* spp. to antimony (Hadighi et al. 2006, Pourmohammadi et al. 2011).

We aimed in this study, to establish a feasible and suitable approach for detecting resistant strains at the clinical level for implementation of a rational therapy and mapping the prevalence of resistance in Fars Province, south part of Iran, evaluating these resistance isolates in the in vitro assay, and determining if this test could be extendable to the in vivo assay, which has not been applied to *L. major* yet.

## Materials and Methods

### Clinical isolates

Forty-three field strains enrolled in this study were collected previously from patients infected with *L. major* in Fars Province between 2009 and 2010. The study was conducted under full respect to Ethics Statements and after appropriate informed consent from the patients (Pourmohammadi et al. 2011). Briefly, individuals suspected of ZCL were referred to Valfajr Health Center in Shiraz and infection with *L. major* was confirmed by PCR. Then, patients were treated with intramuscular Glucantime® at 20mg/kg/day dose for 20 days as a standard protocol of WHO. After finishing the treatment course, they were followed up for six weeks and three month. Patients were considered unresponsive to treatment if amastigotes were found on light microscope examination of Geimsa stained slides prepared from the lesion edge.

### Reference sensitive strain

The reference strain (MRHO/IR/75/ER), sensitive to Glucantime® treatment, used in this experiment was originally bought from the Pasture Institute of Iran was. In addition, a clinical isolate, which showed high rate of resistance to Glucantime® in both clinical level and in vitro assay, was used as a reference resistant strain in this work.

### Anti-*Leishmania* drug

Meglumine antimonite (Glucantime®) was prepared from Sigma-Aldrich Chemical Company (011M0125V). The stock solution was produced by dissolving of drug powder in DMEM medium, to reach four different drug dilutions l0. With respect to finding a more suitable drug dose for establishing in vitro assay, which could be more extendable to clinical consequences, we used four different concentrations of Glucantime® obtained through further dilution in complete medium (15 μg/ml, 30 μg/ml, 45 μg/ml and 60 μg/m).

### The mouse macrophage cell line (J774)

This cell line was bought from national cell bank of Pasture Institute of Iran and grew in DMEM medium supplemented with 12% FBS, 100IU/ml of penicillin, and 100 g/ml of streptomycin and incubated at 37 °C under 5% CO_2_ atmosphere until it reached to logarithmic phase of growth.

### The in vitro intracellular susceptibility assay

The efficacy of meglumine antimonite of filed isolates was conducted on the mouse macrophage cell line (J774) as previously described (Faraut-Gambarelli et al. 1997). Briefly, the numbers of 5 × 10^5^ of J774 cells in log phase of growth were seeded in each 8-well chamber slides with cover slips (Nunc, 177445). Then they were left for cells adhesion for 1 h at 37 °C and 5% CO_2_. Adherent macrophages were infected with late stationary phase of promastigotes at a ratio of 10:1 (promastigote/cell) for 4 h. After that, the excess promastigotes, which were not able to infect macrophages, were removed and each well was replaced with 400-μl medium containing Glucantime®. Meanwhile, all experiments were done in triplicate for each drug concentration. There was a control well against three treated wells contained only infected macrophage and 400μl of complete medium without any drug. Lastly, after 5 days incubation, all wells were fixed with absolute methanol and stained with 10% Geimsa for microscopic examination. Finally, parasite burdens as the percentage of infected macrophages × (mean number of amastigotes/macrophage) were calculated and compared to the burdens for the untreated infected control wells.

### Statistical analysis

Based on the reduction of total parasite burdens, 50% inhibitory concentrations (IC50) were calculated for each well. At least, regarding to categorizing strains to resistant and sensitive isolates, IC50 of each isolate was compared with IC50 of reference (sensitive and resistant) isolates by the chi-square test and McNemar. If there was a significant difference between IC50 of reference strains and each field isolate, this clinical strain was characterized as resistant to drug in vitro assay and vice versa. For the determining of correlation between clinical outcomes and in vitro sensitivity test, we used Kappa coefficient that a P< 0.05 was considered statistically significant. Statistical analysis was performed by the use of SPSS version 16.0 (Chicago, IL, USA).

## Results

Out of 43 ZCL patients, 15 cases (34.9%) were non-responsive (non-healing) and 28 (65.1%) were responsive (healing) after complete treatment with Glucantime® at clinical level. In this experiment, IC50 values of field isolates represented ranging from (15 μg/ml to 60 μg/ml), while the IC50 was (˂15 μg/ml) for reference sensitive strain and was (˃60 μg/ml) for reference resistant strain.

The comparative results between clinical outcomes and in vitro sensitivity test are summarized in Table 1. In the highest drug dose (60 μg/ml), all of responsive field strains represented sensitivity profiles in vitro as well, although four of fifteen strains which were determined as resistant at clinical level were sensitive in the in vitro assay at this drug dose. Furthermore, the result of McNemar and Kappa coefficient test (P< 0.05) conducted for finding the sensitivity and specificity and agreement between clinical outcomes and in vitro assay is shown in Table 1. Overall, a best sensitivity, specificity (100/73) and strong correlation (78.9%) was observed especially in terms of using 60μg/ml of Glucantime® between the results of clinical sequence and the in vitro test.

**Table 1.** The comparison between the susceptibility of in vitro and clinical outcomes of *Leishmania major* field isolates to Glucantime® by McNemar and Kappa

**Drug dose**	**In vitro assay**	**Clinical outcomes Sensitive n (%)**	**Clinical outcomes Resistant (%)**	**P-value for McNemar**	**Sensitivity/Specificity**	**Kappa**	**Total**
**15 μg/ml**	Sensitive	20 (71.4)	6 (40)	0.79	71/60	0.35 (.04)	26(60.5)
	Resistant	8 (28.6)	9 (60)				17(39.5)
**30 μg/ml**	Sensitive	26 (92.9)	4 (26.7)	0.69	93/73	0.68 (<0.001)	30(69.8)
	Resistant	2 (7.1)	11 (73.3)				13(30.2)
**45 μg/ml**	Sensitive	28 (100)	6 (40)	.03	100/60	.66 (< 0.001)	34(79.1)
	Resistant	0 (0)	9 (60)				9(29.9)
**60 μg/ml**	Sensitive	28 (100)	4 (26.7)	.125	100/73	.78 (< 0.001)	32(74.4)
	Resistant	0 (0)	11 (73.3)				11(25.6)

## Discussion

Regarding the increasing rate of antimony failures in ZCL patients in Iran, this study was done to set out a reliable in vitro method to determine the antimonial susceptibility of clinical isolates of *L. major* in Fars Province, as there was no report of a comprehensive work on this issue in Iran. In fact, for the first time we aimed to assess this test on *L. major* clinical strains. Since the intracellular amastigote, method had the best agreement with clinical responses rather than using promastigote form, we decided to evaluate the susceptibility of antimony in this stage of parasite as well (Maia et al. 2013, Coelho et al. 2014).

In fact, the current results revealed a strong correlation (coefficient: r= 0.7860) and the best sensitivity, specificity between the clinical outcomes of antimony therapy and the in vitro antimonial susceptibility test. Indeed, this study was conducted on *L. major* clinical strains and has great corroboration with previous researches on various species of *Leishmania* around the world (Fumarola et al. 2004, Vermeersch et al. 2009, Aït-Oudhia et al. 2012, Maia et al. 2013, Coelho et al. 2014). Like our findings, in another research conducted on 26 clinical isolates of *L. tropica* in Iran by Hadighi et al. there was an excellent correlation between clinical outcomes and the in vitro susceptibility test in amastigote-marophage model (Hadighi et al. 2006). Based on these findings, this in vitro assay can be considered as a valid method for evaluation of drug sensitivity in both species of *Lesihmania* causing cutaneous leishmaniasis Iran.

Despite these supportive investigations, however, no potential agreement was noticed between the clinical response of *L. braziliensis*, and *L. donovani* isolates, and the intracellular amasigote in vitro system in studies performed in various endemic regions around the globe (Rojas et al. 2006, Yardley et al. 2006, Rijal et al. 2007). Overall, these agreements and contradictions between different reports can be explained by some factors that may affect the results of the in vitro assays. Firstly, partially distinctive genetic make-ups of *Leishmania* species would be a reasonable proof to clarify these disagreements among several surveys carried out on different species of *Leishmania* (Croft et al. 2006). However, our findings obtained from field strains of *L.major* were relatively in agreement with the results from other species of *Leishmania* (*L. tropica*) in Iran (Hadighi et al. 2006). Moreover, clinical definition of resistance seems to be an important clue for justifying these discrepancies among different studies (Lira et al. 1999, Croft 2001, Rijal et al. 2007). In the present work, the patients were categorized as responsive and unresponsive after six weeks and following three months of a standard therapy protocol; while, in another study by Luz et al. (2009) this period was extended to 6 and 12 months following the therapy. There were also some differences among various studies in the operational protocols such as time of drugging, the promastigote-macrophage ratio, and timing of the cessation of the experiment (da Luz et al. 2009). Obviously, if we applied another approach, perhaps the correlation value would have been changed. Therefore, in this context, we made a big effort to standardize the in vitro procedures through investigating various factors, which could consequently make the results extendable to clinical outcomes.

In the present experiment, out of 15 unresponsive clinical isolates to Glucantime®, 11 cases were also resistant in the in vitro assay. This evidence can be associated clearly with the intrinsic natures of various *Leishmania* strains, which make them show original sensitivity or resistance to antimonial components (Polonio and Efferth 2008, Jeddi et al. 2011). Since *L. major* causes the ZCL disease, it is assumed that these parasites have never been exposed to drugs, and so these 11 clinical isolates which are resistant at both clinical and in vitro levels, are inherently resistance to antimony. Beyond the genetic factors, some researches have focused on revealing the antimony resistant aspects and have demonstrated that host factors such as genetics and particularly the immune status may have crucial roles on the efficacy of antimony on *Leishmania* parasites in vivo (Murray and Delph-Etienne 2000, Campino et al. 2006, Sundar and Goyal 2007). According to this fact, we observed some clinical strains, cured after complete therapy with Glucantime®, while they showed a resistant phenotype in the in vitro susceptibility test. Similar finding was reported by Vanaerschot et al. (2013) on *L. donovani*, and they infered that perhaps the immune components will act in a great synergy with the drug in order to defend more strongly against *Leishmania* cells. Therefore, recognition of some isolates as responsive to therapy at clinical level categorized as resistant in the in vitro assay is more likely due to lack of immune system effects in the in vitro tests. Furthermore, even different strains of one species of *Leishmania* may use multiple resistant mechanisms against antimony components in diverse circumstances, in vitro and in vivo (Ouellette et al. 2004, Chakravarty and Sundar 2010). Therefore, it seems to be a good explanation to describe why some of our isolates showed reversed phenotypes in vitro and at clinical levels. For example, there were some isolates, which were not responsive to therapy at clinical level, while these were responsive in vitro and vice versa. Perhaps they behaved differently and used various anti-drug mechanisms in different conditions.

## Conclusion

In the absence of a gold standard method for monitoring drug sensitivity, based on findings of this paper, it is highly recommended that this intracellular-amastigote in vitro assay can be utilized as a reliable method for both monitoring the antimony efficacy of field strains of *L. major* and epidemiological studies in different endemic areas of Iran. However, with respect to multi factorial phenomenon of antimony resistance in different field strains, further researches should be undertaken with more samples and in other endemic areas of Iran to confirm our preliminary data.
